# Substance use: comparison between the pandemic period and post-pandemic

**DOI:** 10.1192/j.eurpsy.2023.373

**Published:** 2023-07-19

**Authors:** A. H. I. Abu Shehab, T. Mihai, I. D. Bulgaru, V. Gheorman, D. C. Voinescu, A. Ciubară

**Affiliations:** ^1^Psychiatry, “Elisabeta Doamna” psychiatry hospital; ^2^Psychiatry, „Dunărea de Jos” University of medicine, Galati; ^3^Forensic Medicine, University of Medicine and Pharmacy “Grigore T. Popa”, Iasi; ^4^Psychiatry, University of Medicine and Pharmacy of Craiova, Craiova; ^5^Rheumatology, „Dunărea de Jos” University of medicine, Galati, Romania

## Abstract

**Introduction:**

Substance abuse is a pattern of compulsive substance use that is accompanied by repeated substantial interpersonal, social, professional, or legal negative effects, such as repeated absences from work or school, arrests, or marital issues.

**Objectives:**

The study aims to show the influence of psychological factors among the consumption of alcohol in the general population.

**Methods:**

A retrospective study that was conducted at the “Elisabeta Doamna” Psychiatry hospital of Galati, Romania. The study shows the fluctuations in hospital admissions of patients with alcohol related disorders in the period November 2020 till September 2022.

**Results:**

The study was conducted to show the difference in the number of admissions before and after the date of 1^st^ March 2022, which is the date when the authorities in Romania have lifted the restrictions that were implemented to reduce the spread of Covid_19 virus. The number of total cases in the period between the 1^st^ of November 2020 and 28^th^ of February 2022 were 672 patients, from which 518 patients were males from Urban areas. In the period between the 1^st^ March 2022 and the 1^st^ of September 2022, the number of patients that were admitted due to substance use were 232 cases. This shows a significant decrease in the number of admissions that can be related to the decrease of psychological stressing factors that were accompanied with the restriction measure of the pandemic.

**Conclusions:**

Overall, the results of this retrospective study suggest that the prevalence of adult substance use has declined with the decrease of the restriction measures that were imposed by the authorities. Monitoring and ongoing surveillance of substance use will be necessary over the years following the pandemic.

**Disclosure of Interest:**

None Declared

